# Investigating Fetuin-A and Paraoxonase-1 Activity as Markers in Polycystic Ovary Syndrome Based on Body Mass Index: A Prospective Case-Control Study

**DOI:** 10.7759/cureus.18553

**Published:** 2021-10-06

**Authors:** Tugba Gurbuz, Sebnem Alanya Tosun, Aysegul Cebi, Oya Gokmen, Murat Usta

**Affiliations:** 1 Department of Obstetrics and Gynaecology, Medistate Kavacik Hospital, Istanbul, TUR; 2 Department of Obstetrics and Gynaecology, Giresun University Faculty of Medicine, Giresun, TUR; 3 Department of Biochemistry, Giresun University Faculty of Health Sciences, Giresun, TUR; 4 Department of Biochemistry, Giresun University Faculty of Medicine, Giresun, TUR

**Keywords:** polycystic ovary syndrome, paraoxonase-1, fetuin-a, body mass index, obesity

## Abstract

Introduction

Polycystic ovary syndrome (PCOS) may have an increased risk for the development of systemic and metabolic pathogenesis such as cardiovascular diseases, insulin resistance (IR), diabetes mellitus (DM) and dyslipidemia. However, there is no reliable marker to show the relation. Fetuin-A is an adipokine whereas paraoxonase-1 (PON-1) is a high-density lipoprotein-linked enzyme to demonstrate oxidative stress. This study aimed to evaluate serum fetuin-A and PON-1 levels in infertile PCOS women based on body mass index (BMI).

Methods

A prospective case-control study in a university setting was designed. A total of 88 patients admitted to the Giresun University Faculty of Medicine Gynecology Clinic between February and April 2021 were included in the study. The subjects were divided as follows: PCOS-low-BMI (BMI≤25) vs. Controls-low-BMI (BMI≤25) and PCOS-high-BMI (BMI>25) vs. Controls-high-BMI (BMI>25). Those who had at least two criteria of the 2003 Rotterdam Consensus were diagnosed with PCOS. Serum fetuin-A and PON-1 levels were compared.

Results

The mean levels of fetuin-A were not significantly different in the groups (p=0.955). Serum PON-1 levels were lower in the PCOS group (109.1±61.4 vs. 140.1±80.0; p=0.040), but it lost significance with adjusted values for covariants as age and BMI. Although PON-1 was not significantly different in the PCOS group of BMI<25 kg/m^2^ subgroup, it was significantly lower in the PCOS group of BMI≥25 kg/m^2^ subgroup (p=0.820 vs. p=0.048).

Conclusion

Serum fetuin-A activity did not differ with PCOS. Serum PON-1 might be a promising and research-worthy marker, especially for obese PCOS patients.

## Introduction

Polycystic ovary syndrome (PCOS) is a frequent endocrinopathy that impacts 5 to 8% of women at reproductive age [1]. It is characterized by polycystic ovaries, hyperandrogenism, and chronic anovulation, and is regarded as a multifactorial disorder because of its genetic and environmental onset [2, 3]. Due to its systemic and metabolic pathogenesis, the risk of cardiovascular diseases, insulin resistance (IR), type II diabetes mellitus (DM), hyperinsulinemia, and dyslipidemia may increase in these patients [4, 5].

Women with PCOS have hyperinsulinemia, which influences the hypothalamic-pituitary-gonadal axis, enhancing the luteinizing hormone (LH) secretion over follicle-stimulating hormone (FSH), reducing follicular maturation and sex hormone-binding globulin (SHBG), and producing ovarian androgen [4]. In addition, women with PCOS are at higher risk of other metabolic diseases such as obesity [6]. Obesity also plays an essential role in the pathophysiology and clinical features of PCOS since it enhances the free androgen circulation in the blood, altering the function of ovarian granulosa cells and developing follicles [7].

When the number of genes related to PCOS increases rapidly, the coactions of multiple genomic variants with environmental factors like obesity may cause PCOS [8]. Central accumulation of body fat increases the waist-to-hip circumference ratio (WHR) and the risk of DM and cardiovascular diseases [9, 10, 11].

Fetuin-A is an adipokine and a hepatokine that can be expressed and secreted by the adipose tissue [12, 13]. Recently, it was found that fetuin-A polymorphisms are related to type 2 DM, and serum fetuin-A levels are reduced, increased, or remain the same in obese patients [14, 15, 16]. There is a conflicting result indicating a relationship between fetuin-A and PCOS in various studies. 

Serum paraoxonase-1 (PON-1) is an enzyme linked with the antioxidant high-density lipoprotein (HDL) codified by the PON-1 gene and is expressed mainly in the liver [17]. Due to the adverse effect of oxidative stress on insulin metabolism, lower levels of serum PON-1 may cause IR due to PCOS [9, 18]. San Millán et al. [8] stated that homozygosity for -108T alleles was more common in the PCOS population than in the normal population and supposed that oxidative stress increased in PCOS women due to lower PON-1 activity.

Although it is known that PCOS is linked to metabolic diseases, it is obvious that new markers showing this condition are strongly needed in clinical practice. The present study aimed to evaluate serum fetuin-A and PON-1 levels as possible markers in infertile women with PCOS based on body mass index (BMI).

## Materials and methods

The prospective case-control study was carried with a total of 88 patients who were admitted to the Giresun University Faculty of Medicine Gynecology and Obstetrics clinic between February and April 2021. This study was approved by the Local Ethics Committee of Giresun University (Date: 18.02.2021, Decision no: 14). All the patients signed the given informed consent.

The case group consisted of volunteers diagnosed with PCOS according to at least two criteria of the 2003 Rotterdam Consensus: hyperandrogenism, ultrasonographic view of polycystic ovaries and oligo/anovulation [19]. The control group consisted of volunteers diagnosed with unexplained infertility. The participants were separated into groups according to BMI: PCOS-low-BMI (BMI≤25) vs. controls-low-BMI (BMI≤25) and PCOS-high-BMI (BMI>25) vs. controls-high-BMI (BMI>25).

Infertile patients who were aged 18-39 and could not conceive despite unprotected intercourse for at least 1 year and were non-smokers were included in the study. The other inclusion criterion for all groups was not to use oral contraceptives or any drugs known to altered hormone, lipid or insulin metabolism for the last 3 months. Smokers, those who were diagnosed with hypertension, diabetes mellitus, hypercortisolism and any other endocrinopathy, women who used medication for polycystic ovary syndrome or used oral contraceptives in the last 3 months, women who had increased insulin sensitivity or used medications for hyperlipidemia, etc. were excluded from the study.

Prolactin, FSH, LH, estradiol (E2), thyroid-stimulating hormone (TSH), free thyroxine (FT4), anti-mullerian hormone (AMH), low-density lipoprotein (LDL), triglyceride, total cholesterol, HDL, glucose, insulin, SHBG and dehydroepiandrosterone sulfate (DHEA-S) values were measured on the third day of their menstrual cycle after 8 hours of fasting. Homeostatic Model Assessment for Insulin Resistance (HOMA-IR) was calculated using the following formula: HOMA-IR = ((fasting insulin (mU/mL) x fasting glucose (mg/dL)) / 405). The remaining serum sample was stored at -80ºC until the measurement of fetuin-A and PON-1 levels.

Serum PON-1 activity was measured with a photometric assay using a PON assay kit purchased from Rel Assay Diagnostics, Turkey. An enzyme-linked immunosorbent assay (ELISA) was used to measure fetuin-A using Human Fetuin-A ELISA kit (Bioassay Technology Laboratory, Co. Ltd., Shanghai, China) as instructed by the manufacturer. Human FETU-A (fetuin-A) antibody plates were used. Samples were added and bound to antibodies on the wells. Then, a biotinylated human FETU-A antibody was added and bound to FETU-A in the sample. Streptavidin-horseradish peroxidase (HRP) was added and bound to biotinylated FETU-A antibody. Unbound streptavidin-HRP has washed away after incubation in the washing step. Then, substrate solution was added, and the color was developed based on the human FETU-A amount. The stop solution terminated the reaction. Absorbance was measured to be 450 nm.

Statistical analysis

The statistical analysis was performed with Number Cruncher Statistical System 2004 (NCSS, LLC, East Kaysville, USA) and GraphPad Prism 9.0.1 (GraphPad Software Inc., San Diego, USA). The suitability of continuous variables to normal distribution was investigated using the Kolmogorov-Smirnov test. Variables showing Gaussian distribution are given as the mean ± SD; variables showing a non-Gaussian distribution were given as the median (25. percentile - 75. percentile). Student's t-test or Mann-Whitney U test was used in independent group comparisons. Pearson's Chi-square test was used for the comparison of group proportions. Correlations between variables were analyzed using Pearson’s correlation coefficient (r). The adjusted mean ± standard error (SE) values obtained by controlling (clearing their effects) with analysis of covariance (ANCOVA) analyses, in which the potential confounders determined by regression analysis were evaluated, were used in group comparisons. Two-way analysis of variance (ANOVA) was used to analyze the difference between the means of more than two groups. Statistical significance was evaluated at the p<0.05 (two-tailed) level.

When the sample size was calculated with the G*Power 3.1 (http://www.gpower.hhu.de/) program, the total mean of four groups compared based on the two-way ANOVA with the effect size of 35%, power of 80% and 0.22 type 1 error, was found to be at least 86 patients. A priori sample size was confirmed as calculating for α=0.05 and β=0.80 (two-tailed hypothesis) using the mean of difference and the SD of the control group from the source study by G*Power 3.1.9.2 [14].

## Results

The demographic, anthropometric, and laboratory parameters of control and PCOS groups were summarized in Table 1. The median values of BMI and HOMA-IR were significantly higher in the PCOS group compared to the control group, but the mean age was significantly lower. In addition, the median values of total/free testosterone, AMH, DHEA-S were significantly higher in the PCOS group, but the median value of SHBG was significantly lower. The mean fetuin-A was not significant difference in the groups, but the PON-1 was significantly lower in the PCOS group compared to the control group.

**Table 1:** Data on demographic, anthropometric, and laboratory parameters of the study groups The parameters with normal distribution in the groups are shown as mean ± SD, those that do not are shown as median (interquartile range - IQR). PCOS: polycystic ovary syndrome, BMI: body mass index, HOMA-IR: homeostatic model assessment for insulin resistance, HDL: high-density lipoprotein, LDL: low-density lipoprotein, AMH: anti-müllerian hormone, DHEA-S: dehydroepiandrosterone sulfate, SHBG: sex hormone-binding globulin, PON-1: paraoxonase-1.

	PCOS Group (n=50)	Control Group (n=40)	p
Age, years	24.8 ± 4.8	31.3 ± 5.6	< 0.0001
BMI, kg/m^2^	28.4 ± 6.7	25.9 ± 4.6	= 0.047
BMI <25 kg/m^2^, n (%)	19 (38.0%)	20 (50.0%)	= 0.254
BMI ≥ 25 kg/m^2^, n (%)	31 (62.0%)	20 (50.0%)	= 0.254
Smoking, n (%)	13 (26.0%)	10 (25.0%)	= 0.914
Waist circumference, cm	91.6 ± 16.8	88.1 ± 11.0	= 0.261
Hip circumference, cm	110.0 ± 15.0	106.5 ± 10.0	= 0.215
Waist /Hip circumference	0.83 ± 0.07	0.83 ± 0.06	= 0.842
Glucose, mg/dL	96.7 ± 9.9	95.7 ± 7.3	= 0.585
Insulin, µU/mL	16.0 (11.1 – 26.5)	11.7 (9.0 – 13.7)	= 0.002
HOMA-IR	3.93 (2.58 – 6.78)	2.66 (2.10 - 3.33)	= 0.038
Total cholesterol, mg/dL	174.8 ± 42.8	176.9 ± 40.6	= 0.815
Triglycerides, mg/dL	89 (64 – 156)	82 (61 – 119)	= 0.310
HDL-cholesterol, mg/dL	51.6 ± 13.2	52.4 ± 9.7	= 0.752
LDL-cholesterol, mg/dL	103.4 ± 31.8	102.0 ± 37.5	= 0.853
Total Testosterone, ng/mL	0.47 (0.30 – 0.61)	0.21 (0.13 – 0.29)	< 0.0001
Free Testosterone, pg/mL	3.30 (2.10 – 4.44)	1.72 (0.15 – 2.50)	< 0.0001
AMH, ng/mL	7.2 ± 4.0	2.8 ± 1.8	< 0.0001
DHEA-S, ng/mL	277.7 (218.1 -351.3)	246.8 (188.7 – 272.0)	= 0.046
SHBG, nmol/L	33.7 (21.4 – 50.1)	49.8 (38.3 – 59.0)	= 0.006
Fetuin-A, µg/mL	1298.0 ± 429.1	1292.4 ± 501.2	= 0.955
PON-1, U/L	109.1 ± 61.4	140.1 ± 80.0	= 0.040

The HOMA-IR levels of the study population showed an asymmetric profile, and the variance-stabilizing Ln-transformation (natural logarithm-transformed HOMA-IR) of HOMA-IR was used in the subsequent analyzes.

The results of ANCOVA analyses, in which the demographic, anthropometric, and laboratory parameters found to be significantly differenced in the groups were evaluated as covariant, are shown in Tables 2, 3. The adjusted means of fetuin-A and PON-1 (controlling for the covariates ‘age, BMI, and Ln-transformed HOMA-IR’) were not statistically significant between groups. These results showed that these covariants did not have a confounding effect on fetuin-A levels in the groups. But especially age as a covariant had a remarkable confounding effect on PON-1 levels in the groups.

**Table 2:** The adjusted mean ± SE values and comparison results of fetuin-A levels (µg/mL) (controlling for the covariates ‘age and Ln-transformed HOMA-IR’) in PCOS and control groups Age, BMI and Ln-transformed HOMA-IR were evaluated at 27.70 years, 27.24 kg/m2 and 1.180, respectively. PCOS: polycystic ovary syndrome, BMI: body mass index, HOMA-IR: homeostatic model assessment for insulin resistance, SE: standard error, Ln: natural logarithm.

	PCOS Group	Control Group	p
Not controlled for covariants	1298.0 ± 60.7	1292.4 ± 79.3	= 0.955
Controlling for age	1285.3 ± 71.3	1308.3 ± 81.3	= 0.844
Controlling for BMI	1300.6 ± 66.4	1289.1 ± 74.4	= 0.910
Controlling for Ln-transformed HOMA-IR	1270.2 ± 66.1	1327.1 ± 74.3	= 0.579

**Table 3:** The adjusted mean ± SE values and comparison results of PON-1 levels (U/L) (controlling for the covariates ‘age and Ln-transformed HOMA-IR’) in PCOS and control groups Age, BMI and Ln-transformed HOMA-IR were evaluated at 27.70 year, 27.24 kg/m2 and 1.180, respectively. PCOS: polycystic ovary syndrome, BMI: body mass index, HOMA-IR: homeostatic model assessment for insulin resistance, PON-1: paraoxonase-1, SE: standard error, Ln: natural logarithm.

	PCOS Group	Control Group	p
Not controlled for covariants	109.1 ± 8.7	140.1 ± 12.6	= 0.040
Controlling for age	110.6 ± 12.4	138.3 ± 12.4	= 0.123
Controlling for BMI	110.4 ± 10.0	138.6 ± 11.3	= 0.068
Controlling for Ln-transformed HOMA-IR	110.6 ± 10.2	138.3 ± 11.5	= 0.084

BMI was more slightly positively correlated with age (r=0.257, p=0.015). Considering this situation, for PON-1 levels, the interaction between PCOS and overweight was found to be significant with two-way variance analyzes (Table 4). The means of PON-1 were not significantly different in the study groups of BMI<25 kg/m2 subgroup and were significantly lower in the PCOS group of BMI≥25 kg/m2 subgroup. In addition, the interaction between PCOS and overweight was not significant for the means of age.

**Table 4:** Results of two-way ANOVA analysis for the PON-1 levels (U/L) in BMI subgroups of PCOS and control groups Two-way interaction was defined as polycystic ovary syndrome*overweight. PCOS: polycystic ovary syndrome, BMI: body mass index, HOMA-IR: homeostatic model assessment for insulin resistance, PON-1: paraoxonase-1, ANOVA: analysis of variance.

Subgroups	PCOS Group (n=50)	Control Group (n=40)	Subgroups significance (p)	Two-way interaction significance (p)
HOMA-IR < 2.5	112.4 ± 57.9	138.2 ± 76.7	= 0.351	= 0.820
HOMA-IR ≥ 2.5	108.2 ±63.1	141.5 ± 83.9	= 0.081	
BMI <25 kg/m^2^	124.0 ± 57.9	122.1 ± 55.1	= 0.917	= 0.048
BMI ≥ 25 kg/m^2^	100.0 ± 62.6	158.1 ± 97.0	= 0.012	

## Discussion

PCOS is strongly considered to be associated with metabolic syndrome. Also, obesity is one of the risk factors for unfavorable metabolic dysfunction. Currently, there is no routinely used biomarker for screening PCOS patients for metabolic syndrome. In the present study, we compared serum fetuin-A and PON-1 levels in PCOS and control groups based on BMI to find out a new reliable biomarker. The findings showed that the fetuin-A levels were not altered with PCOS. Although PON-1 level were not significant difference in the normal weighted PCOS group, it was significantly lower in the obese PCOS group. When the effect of age and BMI between the compared groups is removed, PON-1 level was still low in the PCOS group, but it is not statistically significant.

Dursun et al. stated that PCOS reduced serum PON-1 level and this may be related to the probability of increased atherosclerotic heart diseases [9]. However, this is not consistent with the study by San Millan et al., who did not show a significant difference between the PCOS and control groups in the serum PON-1 activities [20]. Xita et al. found no association between PON-1 and PON-2 gene polymorphisms and PCOS but the association between the L55M polymorphism and insulin levels, which may be implicated in the PCOS's IR phenotype [21]. Mohamed et al. found an association between -108TT and -55MM genotypes and increased BMI in PCOS and the controls and the effect of the PON1-108C/T and L55M polymorphisms on the development of PCOS, which is consistent with our study results [22].

There are some studies demonstrated the association between PCOS and oxidative stress and hyperinsulinemia [23-25]. ElSirgany et al. showed that the PCOS group had a significantly higher fetuin-A concentration than the control group and found a correlation between fetuin-A level and other hormones measured in PCOS women who especially sought fertility [26]. Aghilla et al. also found similar results while the present study did not show any significant difference between the two groups in fetuin-A [27]. Liu et al. found that Fetuin-A had a positive correlation with BMI and was associated with PCOS and IR, ovarian hyperandrogenism, and dyslipidemia in PCOS women, which was in line with the study by Enli et al., who found an association between Fetuin-A level and IR in PCOS women [28, 29]. Abali et al. found higher fetuin-A levels in euglycemic patients with PCOS [30]. Gulhan et al. revealed that both groups did not show a significant difference between the two groups in fetuin-A levels, which is consistent with our results [31].

Kozakowski et al. found that fetuin-A level increased significantly in overweight or obese PCOS women but was not different among normal obese/overweight women, while our study did not support this relation [32]. As Kozakowski et al., similar results were reported by Liu et al. [28]. Further studies are needed to distinguish whether the elevation of fetuin-A is really due to adipose tissue or whether it may be elevated by insulin resistance or hyperandrogenism. 

Patients with PCOS are known to have abnormal lipid profiles like higher levels of cholesterol, triglycerides and low-density lipoprotein (LDL) and lower levels of total HDL [9]. However, in the present study, there are comparable HDL levels in both groups. It is known that PON-1 supplies anti-oxidative modification of HDL and is useful to avoid from the oxidant activity of LDL [18]. Although PON-1 activity was lower in obese PCOS group, no difference was seen in lean PCOS group and for adjusted values. This lack of difference may be due to the comparable HDL levels.

To our knowledge, this is the first study comparing fetuin-A and PON-1 groups according to BMI in PCOS patients. We have revealed those lower serum PON-1 levels in obese PCOS patients may be a reliable marker to show metabolic dysfunction. Further studies in larger cohorts with more sensitive tests are needed to prove the specific activity of serum PON-1 in obese PCOS patients. 

## Conclusions

Fetuin-A does not appear to be a possible marker for PCOS patients of any weight. PON-1 decreased significantly in the obese PCOS patients. Obesity plays an essential role in the occurrence of PCOS and may affect the PON-1 levels. PON-1 levels may be a possible marker for PCOS among obese women. Further studies are needed to investigate PON-1 levels in PCOS women with different BMIs.

## References

[1] Azziz R, Woods KS, Reyna R, Key TJ, Knochenhauer ES, Yildiz BO (2004). The prevalence and features of the polycystic ovary syndrome in an unselected population. J Clin Endocrinol Metab.

[2] (2004). Revised 2003 consensus on diagnostic criteria and long-term health risks related to polycystic ovary syndrome. Fertil Steril.

[3] Zawadzki JK, Dunaif A (1992). Diagnostic criteria for polycystic ovary syndrome: towards a rationale approach. Polycystic ovary syndrome.

[4] Nahar K, Mahfuza G, Begum SA, Khatun K, Islam MR (2017). Clinical, biochemical and hormonal profile of polycystic ovary syndrome. J Nat Inst Neurosci Bangladesh.

[5] Xu X, Shi Y, Cui Y, Ma J, Che L, Chen ZJ (2012). Endocrine and metabolic characteristics of polycystic ovary syndrome in Chinese women with different phenotypes. Clin Endocrinol (Oxf).

[6] Kelley AS, Smith YR, Padmanabhan V (2019). A narrative review of placental contribution to adverse pregnancy outcomes in women with polycystic ovary syndrome. J Clin Endocrinol Metab.

[7] Pasquali R, Gambineri A, Pagotto U (2006). The impact of obesity on reproduction in women with polycystic ovary syndrome. BJOG.

[8] San Millán JL, Cortón M, Villuendas G, Sancho J, Peral B, Escobar-Morreale HF (2004). Association of the polycystic ovary syndrome with genomic variants related to insulin resistance, type 2 diabetes mellitus, and obesity. J Clin Endocrinol Metab.

[9] Dursun P, Demirtaş E, Bayrak A, Yarali H (2006). Decreased serum paraoxonase 1 (PON1) activity: an additional risk factor for atherosclerotic heart disease in patients with PCOS?. Hum Reprod.

[10] de Groot PC, Dekkers OM, Romijn JA, Dieben SW, Helmerhorst FM (2011). PCOS, coronary heart disease, stroke and the influence of obesity: a systematic review and meta-analysis. Hum Reprod Update.

[11] Jialal I, Devaraj S, Bettaieb A, Haj F, Adams-Huet B (2015). Increased adipose tissue secretion of Fetuin-A, lipopolysaccharide-binding protein and high-mobility group box protein 1 in metabolic syndrome. Atherosclerosis.

[12] Sujana C, Huth C, Zierer A (2018). Association of fetuin-A with incident type 2 diabetes: results from the MONICA/KORA Augsburg study and a systematic meta-analysis. Eur J Endocrinol.

[13] Gambineri A, Pelusi C, Vicennati V, Pagotto U, Pasquali R (2002). Obesity and the polycystic ovary syndrome. Int J Obes Relat Metab Disord.

[14] Abali R, Celik C, Tasdemir N (2013). The serum protein α2-Heremans-Schmid glycoprotein/fetuin-aconcentration and carotid intima-media thickness in women with polycystic ovary syndrome. J Eur J Obstet Gynecol Reprod Biol.

[15] Siddiq A, Lepretre F, Hercberg S, Froguel P, Gibson F (2005). A synonymous coding polymorphism in the alpha2-Heremans-schmid glycoprotein gene is associated with type 2 diabetes in French Caucasians. Diabetes.

[16] Yoo HJ, Choi KM (2015). Hepatokines as a link between obesity and cardiovascular diseases. Diabetes Metab J.

[17] Yang D, Li N, Ma A (2021). Identification of potential biomarkers of polycystic ovary syndrome via integrated bioinformatics analysis. Reprod Sci.

[18] Bin Ali A, Zhang Q, Lim YK, Fang D, Retnam L, Lim SK (2003). Expression of major HDL-associated antioxidant PON-1 is gender dependent and regulated during inflammation. Free Radic. Biol. Med.

[19] Rudich A, Kozlovsky N, Potashnik R, Bashan N (1997). Oxidant stress reduces insulin responsiveness in 3T3-L1 adipocytes. Am J Physiol.

[20] San Millán JL, Alvarez-Blasco F, Luque-Ramírez M, Botella-Carretero JI, Escobar-Morreale HF (2006). The PON1-108C/T polymorphism, and not the polycystic ovary syndrome, is an important determinant of reduced serum paraoxonase activity in premenopausal women. Hum Reprod.

[21] Xita N, Lazaros L, Georgiou I, Tsatsoulis A (2011). Effect of paraoxonase genes on metabolic profile of women with polycystic ovary syndrome. Endocrine Abstracts.

[22] Mohamed AA, Rashed LA, Salam RF (2009). Effect of paraoxonase gene polymorphisms on paraoxonase levels and insulin resistance index in women with polycystic ovary syndrome. Aust J Basic Appl Sci.

[23] Guastella E, Longo RA, Carmina E (2010). Clinical and endocrine characteristics of the main polycystic ovary syndrome phenotypes. Fertil Steril.

[24] Yeon Lee J, Baw C-K, Gupta S, Aziz N, Agarwal A (2010). Role of oxidative stress in polycystic ovary syndrome. Curr Womens Health Rev.

[25] Macut D, Bjekić-Macut J, Savić-Radojević A (2013). Dyslipidemia and oxidative stress in PCOS. Front Horm Res.

[26] ElSirgany S, Badawi H, El-Khayat Z (2019). Serum fetuin a level: a new possible marker for polycystic ovarian syndrome in women with infertility. J Obstet Gynaecol Res.

[27] Aghilla M, Adya R, Tan B, Lehnert H, Ashawesh K, Randeva H (20121). The hepatokine fetuin-a is increased in PCOS women. Association with metabolic syndrome and regulation by metformin. Endocrine Abstracts.

[28] Liu S, Hu W, He Y (2020). Serum fetuin-A levels are increased and associated with insulin resistance in women with polycystic ovary syndrome. BMC Endocr Disord.

[29] Enli Y, Fenkci SM, Fenkci V, Oztekin O (2013). Serum fetuin-A levels, insulin resistance and oxidative stress in women with polycystic ovary syndrome. Gynecol Endocrinol.

[30] Abali R, Celik C, Tasdemir N, Guzel S, Alpsoy S, Yuksel A, Celik E (2013). The serum protein α2-Heremans-Schmid glycoprotein/fetuin-a concentration and carotid intima-media thickness in women with polycystic ovary syndrome. Eur J Obstet Gynecol Reprod Biol.

[31] Gulhan I, Bozkaya G, Oztekin D, Uyar I, Kebapcilar AG, Pamuk B (2012). Serum fetuin-A levels in women with polycystic ovary syndrome. Arch Gynecol Obstet.

[32] Kozakowski J, Jeske W, Zgliczyński W (2014). Fetuin-A levels in lean and obese women with polycystic ovary syndrome. Endokrynol Pol.

